# Comparison of eptinezumab 300 mg with 100 mg for the treatment of migraine: a meta-analysis of randomized controlled studies

**DOI:** 10.4314/ahs.v24i3.43

**Published:** 2024-09

**Authors:** Jing Wang, Xingchuan Li, Zhiguo Yang, Yang Baowang, Ni Zhang

**Affiliations:** 1 Department of Children's Intensive Care Medicine, The Second Hospital of Lanzhou University, Lanzhou, Gansu Province 730000; 2 Department of Gastroenterology, The Second Hospital of Lanzhou University, Lanzhou, Gansu

**Keywords:** Eptinezumab, migraine, randomized controlled trials

## Abstract

**Introduction:**

The efficacy and safety of eptinezumab 300 mg versus 100 mg for migraine remains debatable. We conduct this meta-analysis to compare eptinezumab 300 mg with 100 mg on the treatment of migraine.

**Methods:**

We have searched PubMed, EMbase, Web of science, EBSCO, and Cochrane library databases through April 2021 for randomized controlled trials (RCTs) assessing the effect of eptinezumab 300 mg versus 100 mg on treatment efficacy and safety in migraine patients. This meta-analysis was performed using the random-effect model.

**Results:**

Four RCTs were included in the meta-analysis. Overall, compared with eptinezumab 100 mg in migraine patients, eptinezumab 300 mg was associated with substantially improved 75% responder rate (OR=1.34; 95% CI=1.06 to 1.69; P=0.01), but demonstrated similar monthly migraine days (MD=-0.09; 95% CI=-0.20 to 0.01; P=0.08), 100% responder rate (OR=1.38; 95% CI=0.94 to 2.02; P=0.10), 50% responder rate (OR=1.20; 95% CI=0.97 to 1.48; P=0.10), migraine 1 day after dosing (OR=0.92; 95% CI=0.72 to 1.18; P=0.52), adverse events (OR=1.13; 95% CI=0.77 to 1.65; P=0.53), nasopharyngitis (OR=1.26; 95% CI=0.74 to 2.14; P=0.40), upper respiratory tract infection (OR=1.25; 95% CI=0.83 to 1.88; P=0.29), sinusitis (OR=1.78; 95% CI=0.95 to 3.33; P=0.07) or nausea (OR=1.26; 95% CI=0.68 to 2.32; P=0.46).

**Conclusions:**

Eptinezumab 300 mg may have better efficacy for migraine patients than eptinezumab 100 mg.

## Introduction

As one common, disabling neurologic disorder, migraine results in significantly great disability, high rates of comorbidity, great direct and indirect costs 1,2. Previous study reported that one-year period prevalence of migraine in adults was approximately 10%–15% in Europe and USA 3 4. Migraine attack is a throbbing or pulsating headache, and may cause nausea, vomiting, sensitivity to light or sound 5,6. However, the diagnosis and treatment rates for migraine are low, and migraine preventive treatment is frequently discontinued 7-10.

Currently, calcitonin gene-related peptide (CGRP)-targeted monoclonal antibodies are approved to prevent migraine 11. Blockade of the CGRP pathway is found to be an established method in the acute and preventive treatment of migraine 12. Eptinezumab is a monoclonal antibody that can bind to the CGRP ligand 13. In 476 patients with migraine, eptinezumab was reported to associated with significantly improved patient-reported outcomes (e.g. headache pain freedom) 14. PROMISE-2 study revealed that both the 100 and 300 mg doses of eptinezumab resulted in significant reductions in monthly migraine days, 75% and 50% responder rates in migraine patients 11.

Recently, several studies have compared the efficacy and safety of eptinezumab 300 mg and 100 mg for migraine patients, but the results are conflicting 15-17. This systematic review and meta-analysis of RCTs aims to assess the efficacy and safety of anti-CGRP antibody eptinezumab 300 mg versus 100 mg in migraine patients.

## Materials and methods

This meta-analysis was performed based on the guidance of the Preferred Reporting Items for Systematic Reviews and Meta-analysis statement and Cochrane Handbook for Systematic Reviews of Interventions 18,19. No ethical approval and patient consent were required because all analyses were based on previous published studies.

### Literature search and selection criteria

We have systematically searched several databases including PubMed, EMbase, Web of science, EBSCO, and the Cochrane library from inception to April 2021 with the following keywords: “eptinezumab” AND “migraine”. The reference lists of retrieved studies and relevant reviews were also hand-searched and the process above was performed repeatedly in order to include additional eligible studies.

The inclusion criteria were presented as follows: (1) study design was RCT, (2) patients were diagnosed with migraine, and (3) intervention treatments were eptinezumab 300 mg versus eptinezumab 100 mg.

### Data extraction and outcome measures

Some baseline information was extracted from the original studies, and they included first author, number of patients, age, female, duration of migraine and detail methods in two groups. Data were extracted independently by two investigators, and discrepancies were resolved by consensus. The primary outcomes were monthly migraine days and 75% responder rate. Secondary outcomes included 100% responder rate, 50% responder rate, migraine 1 day after dosing, adverse events, nasopharyngitis, upper respiratory tract infection, sinusitis and nausea.

### Quality assessment in individual studies

The methodological quality of each RCT was assessed by the Jadad Scale which consisted of three evaluation elements: randomization (0-2 points), blinding (0-2 points), dropouts and withdrawals (0-1 points) 20. One point would be allocated to each element if they were conducted and mentioned appropriately in the original article. The score of Jadad Scale varied from 0 to 5 points. An article with Jadad score≤2 was considered to be of low quality. The study had high quality if Jadad score≥3 21.

### Statistical analysis

We assessed mean difference (MD) with 95% confidence interval (CI) for continuous outcomes and odd ratio (OR) with 95% CI for dichotomous outcomes. Heterogeneity was evaluated using the I2 statistic, and I2 > 50% indicated significant heterogeneity 22. The random-effects model was used for all meta-analysis. We searched for potential sources of heterogeneity when encountering significant heterogeneity. Sensitivity analysis was performed to detect the influence of a single study on the overall estimate via omitting one study in turn or performing the subgroup analysis. Owing to the limited number (<10) of included studies, publication bias was not assessed. Results were considered as statistically significant for P <0.05. All statistical analyses were performed using Review Manager Version 5.3 (The Cochrane Collaboration, Software Update, Oxford, UK).

## Results

### Literature search, study characteristics and quality assessment

**Figure 1:**
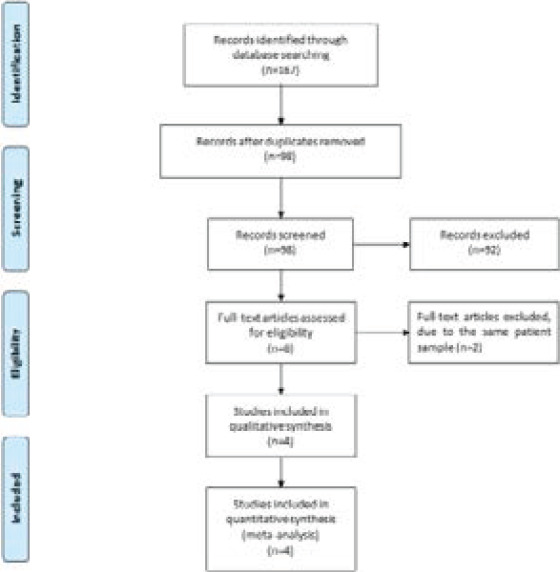
Flow diagram of study searching and selection process

Figure 1 showed the detail flowchart of the search and selection results. 167 potentially relevant articles were identified initially. 69 duplicates and 92 papers after checking the titles/abstracts were excluded. Two studies were removed because of the study design and four RCTs were finally included in the meta-analysis 11, 15-17.

The baseline characteristics of four included RCTs were shown in Table 1. These studies were published between 2019 and 2020. Two RCTs reported the same patient sample, but with different outcomes and treatment duration 11,15. The total sample size was 1396. Among four included RCTs, treatment comparison was set as eptinezumab 300 mg every 12 weeks versus eptinezumab 100 mg every 12 weeks.

**Table 1:** Characteristics of included studies

NO. Author	Eptinezumab 300 mg group					Eptinezumab 100 mg group					Jada scores
	Number	Age (years)	Female (n)	Duration of migraine (y)	Methods	Number	Age (years)	Female (n)	Duration of migraine (y)	Methods	
1 Silberstein 2020	350	41.0 (10.4)	314	19.0 (11.5)	eptinezumab 300 mg administered on day 0 and week 12	356	41.0 (11.7)	307	18.3 (12.2)	eptinezumab 100 mg administered on day 0 and week 12	5
2 Lipton 2020	350	41.0 (10.4)	314	19.0 (11.5)	eptinezumab 300 mg administered on day 0 and week 12	356	41.0 (11.7)	307	18.3 (12.2)	eptinezumab 100 mg administered on day 0 and week 12	5
3 Ashina 2020	224	40.2 (11.72)	199	18.2 (11.75)	eptinezumab 300 mg for up to four doses administered every 12 weeks	223	40.0 (10.66)	179	17.4 (11.18)	eptinezumab 100 mg for up to four doses administered every 12 weeks	4
4 Dodick 2019	121	37.2 (10.0)	104	18.8 (9.9)	a single dose of eptinezumab 300 mg	122	36.7 (9.4)	98	17.4 (10.8)	a single dose of eptinezumab 100 mg	4

Among four included RCTs, three trials reported monthly migraine days, 75% responder rate, 50% responder rate, migraine 1 day after dosing, adverse events, nasopharyngitis, upper respiratory tract infection, sinusitis and nausea 15-17, and two trials reported 100% responder rate 11,17. Jadad scores of the four included studies varied from 4 to 5, and all four studies had high quality based on the quality assessment.

### Primary outcomes: monthly migraine days and 75% responder rate

**Figure 2:**
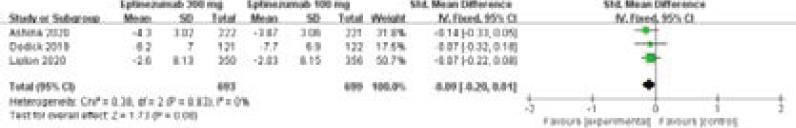
Forest plot for the meta-analysis of monthly migraine days

**Figure 3:**
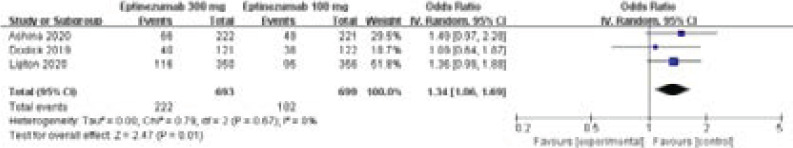
Forest plot for the meta-analysis of 75% responder rate

The random-effect model was used for the analysis of primary outcomes. The results found that compared to eptinezumab 100 mg in migraine patients, eptinezumab 300 mg demonstrated similar monthly migraine days (MD=-0.09; 95% CI=-0.20 to 0.01; P=0.08) with no heterogeneity among the studies (I^2^=0%, heterogeneity P=0.08, Figure 2), but resulted in significantly increased 75% responder rate (OR=1.34; 95% CI=1.06 to 1.69; P=0.01) with no heterogeneity among the studies (I^2^=0%, heterogeneity P=0.67, Figure 3).

### Sensitivity analysis

There was no heterogeneity for the primary outcomes, and thus we did not perform the meta-analysis via omitting one study or subgroup analysis to detect the heterogeneity.

### Secondary outcomes

**Figure 4:**
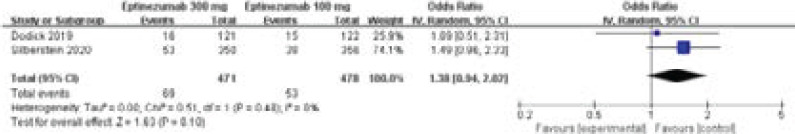
Forest plot for the meta-analysis of 100% responder rate

**Figure 5:**
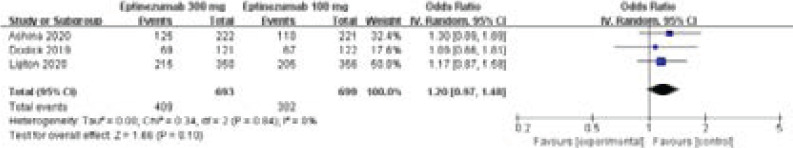
Forest plot for the meta-analysis of 50% responder rate

**Figure 6:**
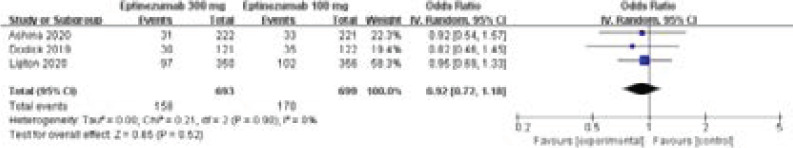
Forest plot for the meta-analysis of migraine 1 day after dosing

**Figure 7:**
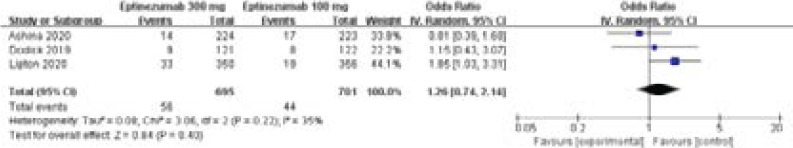
Forest plot for the meta-analysis of adverse events

**Figure 8:**
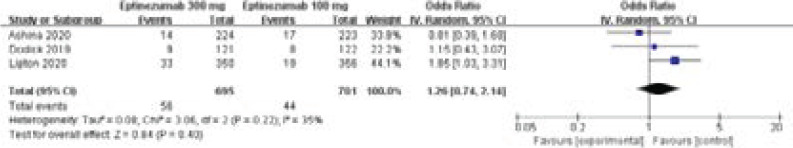
Forest plot for the meta-analysis of nasopharyngitis

**Figure 9:**
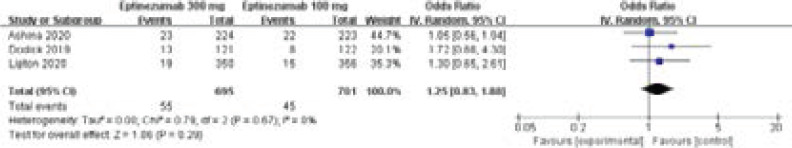
Forest plot for the meta-analysis of upper respiratory tract infection

**Figure 10:**
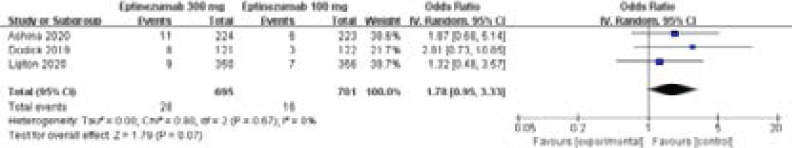
Forest plot for the meta-analysis of sinusitis

**Figure 11:**
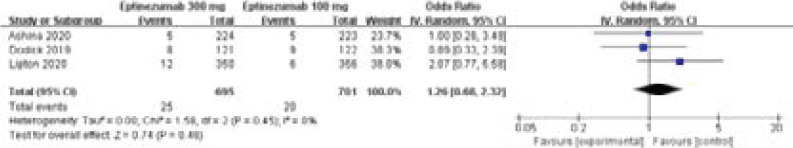
Forest plot for the meta-analysis of nausea

In comparison with eptinezumab 100 mg in migraine patients, eptinezumab 300 mg showed comparable 100% responder rate (OR=1.38; 95% CI=0.94 to 2.02; P=0.10; Figure 4), 50% responder rate (OR=1.20; 95% CI=0.97 to 1.48; P=0.10; Figure 5), migraine 1 day after dosing (OR=0.92; 95% CI=0.72 to 1.18; P=0.52; Figure 6), adverse events (OR=1.13; 95% CI=0.77 to 1.65; P=0.53; Figure 7), nasopharyngitis (OR=1.26; 95% CI=0.74 to 2.14; P=0.40; Figure 8), upper respiratory tract infection (OR=1.25; 95% CI=0.83 to 1.88; P=0.29; Figure 9), sinusitis (OR=1.78; 95% CI=0.95 to 3.33; P=0.07; Figure 10) and nausea (OR=1.26; 95% CI=0.68 to 2.32; P=0.46; Figure 11).

## Discussion

Migraine is one of the most common type among the various causes of headache 23-25, but less than one-third of them are estimated to have consistently effective results with their current treatment 3,26. Many patients still lack effective regimen to reduce the frequency and severity of their migraine attacks despite recent developments in preventive medications for migraine 27,28. Given the results of different dosage regimens of eptinezumab for migraine patients, the dosage regimens of eptinezumab 100 mg and 300 mg showed both significant efficacy for migraine 15,16.

From the perspective of the outcome related to 75% responder rate in our meta-analysis, 300 mg eptinezumab resulted in remarkably better efficacy for migraine patients than 100 mg eptinezumab, but the dosage regimens of eptinezumab 100 mg and 300 mg demonstrated similar monthly migraine days, 100% responder rate, 50% responder rate or migraine 1 day after dosing. These suggested that 300 mg eptinezumab may be more effective to treat migraine patients than 100 mg eptinezumab.

Eptinezumab treatment at the dose of 300 mg and 100 mg was associated with reductions in days of acute medication use, which was an important benefit because overuse of acute medication may result in headache exacerbation, side effects and cost 15,29. Reduction in acute medication also increased the patient acceptance of preventive therapy 30. Mean acute medication days were found to be similar between eptinezumab 300 mg and 100 mg 15. Eptinezumab was well tolerated, and the most of adverse events were mild or moderate 15,31. Eptinezumab 300 mg and 100 mg led to similar incidence of adverse events, nasopharyngitis, upper respiratory tract infection, sinusitis and nausea based on the results of our meta-analysis. One recent pooled analysis pooled analysis of 5 clinical trials confirmed the favourable safety and tolerability profile of eptinezumab (10-1000 mg) for migraine patients 32.

Several limitations exist in this meta-analysis. Firstly, our analysis is based on only four RCTs, and more RCTs with large sample size should be conducted to explore this issue. Next, although there is no heterogeneity, different treatment duration and severity of migraine may produce some bias. Finally, the type of migraine such as chronic or acute migration may affect efficacy evaluation, but it is not feasible to perform their subgroup analysis based on current included RCTs.

## Conclusion

Eptinezumab 300 mg may be more effective to prevent migraine than eptinezumab 100 mg, and more studies should be conducted to explore this issue.
